# Modeling ischemic stroke in a triculture neurovascular unit on-a-chip

**DOI:** 10.1186/s12987-021-00294-9

**Published:** 2021-12-14

**Authors:** Nienke R. Wevers, Arya Lekshmi Nair, Tania M. Fowke, Maria Pontier, Dhanesh G. Kasi, Xandor M. Spijkers, Charlie Hallard, Gwenaëlle Rabussier, Remko van Vught, Paul Vulto, Helga E. de Vries, Henriëtte L. Lanz

**Affiliations:** 1 MIMETAS BV, Leiden, The Netherlands; 2 Department of Molecular Cell Biology and Immunology, Amsterdam UMC, Location VUmc, Amsterdam Neuroscience, Amsterdam, The Netherlands; 3 Department of Translational Neuroscience, University Medical Center Utrecht Brain Center, Utrecht University, Utrecht, The Netherlands; 4 CARIM School of Cardiovascular Diseases, Faculty of Health, Medicine, and Life Sciences, Maastricht University, Maastricht, The Netherlands

**Keywords:** Blood–brain barrier, Neurovascular unit, Stroke, Microfluidics, BBB-on-a-chip

## Abstract

**Background:**

In ischemic stroke, the function of the cerebral vasculature is impaired. This vascular structure is formed by the so-called neurovascular unit (NVU). A better understanding of the mechanisms involved in NVU dysfunction and recovery may lead to new insights for the development of highly sought therapeutic approaches. To date, there remains an unmet need for complex human in vitro models of the NVU to study ischemic events seen in the human brain.

**Methods:**

We here describe the development of a human NVU on-a-chip model using a platform that allows culture of 40 chips in parallel. The model comprises a perfused vessel of primary human brain endothelial cells in co-culture with induced pluripotent stem cell derived astrocytes and neurons. Ischemic stroke was mimicked using a threefold approach that combines chemical hypoxia, hypoglycemia, and halted perfusion.

**Results:**

Immunofluorescent staining confirmed expression of endothelial adherens and tight junction proteins, as well as astrocytic and neuronal markers. In addition, the model expresses relevant brain endothelial transporters and shows spontaneous neuronal firing. The NVU on-a-chip model demonstrates tight barrier function, evidenced by retention of small molecule sodium fluorescein in its lumen. Exposure to the toxic compound staurosporine disrupted the endothelial barrier, causing reduced transepithelial electrical resistance and increased permeability to sodium fluorescein. Under stroke mimicking conditions, brain endothelial cells showed strongly reduced barrier function (35-fold higher apparent permeability) and 7.3-fold decreased mitochondrial potential. Furthermore, levels of adenosine triphosphate were significantly reduced on both the blood- and the brain side of the model (4.8-fold and 11.7-fold reduction, respectively).

**Conclusions:**

The NVU on-a-chip model presented here can be used for fundamental studies of NVU function in stroke and other neurological diseases and for investigation of potential restorative therapies to fight neurological disorders. Due to the platform’s relatively high throughput and compatibility with automation, the model holds potential for drug compound screening.

**Supplementary Information:**

The online version contains supplementary material available at 10.1186/s12987-021-00294-9.

## Background

The vasculature of the brain is made up of specialized endothelial cells that form a tight blood–brain barrier (BBB) [1]. The BBB ensures a homeostatic environment for the brain by controlling the entry of molecules from the circulation. The vasculature of the brain is surrounded by perivascular cells that support and maintain healthy BBB functioning. Among these supporting cells are astrocytes and pericytes, which strengthen the inter-endothelial adherens junctions and tight junctions and maintain proper BBB transport function [2, 3]. The entire structure contributing to BBB function is referred to as the neurovascular unit (NVU) and includes brain endothelial cells, astrocytes, pericytes, neurons, oligodendrocytes, microglia, and the basement membrane [4].

The NVU restricts passive diffusion of large, polar substances and potentially neurotoxic molecules into the brain. Only a selection of molecules, such as oxygen and carbon dioxide, can enter freely. Other essential molecules such as nutrients can enter the brain through specialized transporter systems, for example glucose, which enters via the highly expressed glucose transporter 1 (GLUT-1) [5]. Conversely, more lipophilic molecules and metabolic toxins can be actively removed from the brain through efflux transporters. These efflux transporters include P-glycoprotein (P-gp), breast cancer resistance protein (BCRP), and members of the multidrug resistance protein (MRP) family [6]. While the NVU’s barrier is essential for healthy brain functioning, it also poses a major challenge for drug delivery into the brain, as many drugs can’t freely enter the brain or are removed by efflux transporters [7].

NVU dysfunction is observed in many neurological disorders, ranging from neurodegenerative and neuroinflammatory diseases to dysfunction caused by trauma or stroke [8]. Stroke is the second cause of death worldwide and the leading cause of adult disability [9, 10]. Strokes are of either hemorrhagic or ischemic nature [10]. Hemorrhagic stroke is the result of a vessel rupturing and makes up approximately 20% of all stroke cases. The other 80% of strokes are ischemic, resulting from an occlusion of a blood vessel by a thrombus that disrupts blood flow to the brain. The brain has a very high energy demand and is responsible for approximately 20% of the body’s oxygen consumption and 25% of glucose consumption [11, 12]. For this reason, disrupted blood flow to the brain has detrimental effects.

To date, only one therapeutic agent has been approved for ischemic stroke. Tissue plasminogen activator (tPA) can be administered to dissolve the blood clot and restore blood flow to the brain [13]. However, tPA can only be administered during a relatively short time window (< 4.5 h), as later administration can lead to hemorrhages resulting in a poor patient outcome [14]. Furthermore, intravenous tPA administration is often not effective in removing blood clots in the major intracranial arteries, which account for many cases of ischemic stroke [15]. Recently, several studies have found improved clinical outcome when such cases of ischemic stroke were treated with an alternative approach. Blood clot removal via intraarterial therapy, employing mechanical thrombectomy and/or local delivery of a thrombolytic agent, resulted in improved patient outcome [16]. However, many stroke patients are not eligible for intraarterial therapy. Moreover, the therapy only allows for a short time window, like intravenous tPA administration, and can give rise to new blood clot formation. To date, treatment of ischemic stroke remains far from optimal.

The reasons for this lack of success in treating stroke are multifactorial, but one factor may be found in the predominant use of animal models in preclinical studies [17, 18]. While animal models of the brain’s vasculature have proven valuable, they are costly, time consuming, and allow only limited control over experimental conditions. Moreover, animal studies of neurological disease and NVU function often result in limited translational relevance due to interspecies differences, such as differential expression of important BBB transporters and immune signaling molecules [19–21]. For this reason, researchers also studied stroke in vitro, using (1) traditional oxygen–glucose deprivation techniques [22, 23], (2) chemical methods that inhibit the electron transport chain [22, 23], such as rotenone, antimycin-A, or sodium azide, and (3) enzymatic methods employing glucose oxidase, catalase, and 2-deoxyglucose [24, 25]. One study compared all three techniques to model renal ischemia and reported that the use of antimycin-A was most reproducible [26]. Most in vitro stroke studies were performed using relatively simple models employing immortalized cell lines. The use of more complex in vitro models with improved physiological relevance may aid in finding new therapies for ischemic stroke and other neurological diseases.

The first attempts at in vitro NVU modeling started with cultures of primary brain endothelial cells in traditional two-dimensional (2D) culture systems [27, 28]. Aiming to improve physiological relevance and complexity, the first models in Transwell were developed [29, 30]. In this system, brain endothelial cells were cultured on one side of a semi-permeable membrane and supporting cells on the other. Although the Transwell presented a step forward in physiological NVU modeling, the presence of a membrane and the lack of flow and direct cell–cell contact posed limitations.

In response to those limitations, microfluidic platforms made their appearance in the field of NVU modeling [31, 32]. These platforms make use of tissue culture chips comprising small channels that allow the development of layered three-dimensional (3D) cell cultures under flow [31]. After early work with NVU models based on hollow fiber apparatuses [33–35], Booth and colleagues developed the first NVU model in a chip using vertically stacked planar structures made from polydimethylsiloxane (PDMS) [36]. These planar chips held much thinner membranes than the hollow fiber apparatuses, allowing for improved cell–cell contact in co-culture setups. Many others followed similar approaches in subsequent years, using primary cells and cell lines from various species [37–44]. The most recent microfluidic NVU models still show resemblance to the chip reported by Booth et al., but special focus has been placed on all-human models, using primary material [45], or induced pluripotent stem cell (iPSC)-derived cells [46, 47], allowing for potential use in personalized therapies. Lyu et al. recently applied such an NVU model to study ischemic stroke, using a complex co-culture of endothelial cells, astrocytes, pericytes, microglia, and neurons [48].

While many have developed microfluidic platforms for complex NVU modeling, most of these models are very low in throughput and cumbersome to use. There is a large unmet need for higher throughput, more user-friendly platforms that unite microfluidic NVU models with routine experimentation and the possibility of drug candidate evaluation [49]. We previously reported a BBB model in a microfluidic platform that allows culture of 40 chips in parallel, while being compatible with standard laboratory equipment and automation [50, 51]. The model comprised immortalized human brain endothelial cells grown against an extracellular matrix (ECM) gel in co-culture with immortalized human astrocytes and pericytes.

We here report a microfluidic human NVU model that incorporates primary brain endothelial cells, in co-culture with iPSC-derived astrocytes and neurons grown under bidirectional, gravity-driven perfusion. To showcase the model’s use in studying NVU dysfunction, we developed a protocol to mimic stroke. Under stroke mimicking conditions, the NVU on-a-chip cultures showed reduced BBB integrity, mitochondrial membrane potential, and adenosine triphosphate (ATP), which are common features of ischemic stroke. In contrast to many other microfluidic approaches, the high-throughput and pump-free nature of the platform used renders this method suitable for routine experimentation. The NVU on-a-chip model can be used for fundamental studies of the NVU in health and disease as well as for evaluation of drug candidates under disease mimicking conditions.

## Methods

### Cell culture

Primary human brain microvascular endothelial cells (HBMECs, ACBRI 376, Cell Systems) were cultured in T75 flasks (Nunc™ Easy Flask, Sigma, F7552) in Promocell MV-2 medium (Bioconnect, C-22121). HBMECs were used between passage 4 and 10. iPSC-derived human astrocytes (01434, FujiFilm-CDI) were expanded in T75 flasks (734–2705, Corning) coated with GelTrex (A15696-01, Gibco) in medium comprised of DMEM (31966-021, Gibco) supplemented with 10% fetal bovine serum (FBS, F4135, Sigma), 1 × N2 supplement (17502-048, ThermoFisher), and 1% penicillin/streptomycin (P4333, Sigma). Astrocytes were expanded to P4. iPSC-derived neural stem cells (Ax0018, Axol Bioscience) were expanded in T75 flasks (734-2705, Corning) coated with growth factor reduced Matrigel (Matrigel-GFR, 356231, Corning, 80–100 µg/mL) in expansion medium (see Table 1) for up to 6 passages. Cells were subsequently differentiated on Matrigel-GFR coated 12-well plates (100,000 cells/well) in differentiation medium (see Table 1) for 3 weeks to obtain neurons. Resulting neurons were dissociated using StemPro Accutase (A11105-01, Gibco) for 20 min, after which the cells were cryopreserved in large numbers for subsequent seeding in OrganoPlate. All cells were cultured at 37 °C, atmospheric (20%) O_2_, 5% CO_2_ and regularly tested for mycoplasm contamination and found negative.

**Table 1:** Medium for neuronal expansion and differentiation

Reagent	Supplier	Catalogue number	Final concentration
N2B27 medium			
Neurobasal medium	Gibco	21103049	
N2 supplement	Thermo Fisher	17502-048	1×
B27 supplement	Gibco	12587-010	1×
Non-essential amino acids	Gibco	11140050	1%
GlutaMAX™ supplement	Gibco	35050038	1%
Penicillin-streptomycin	Sigma	P4333	1%
2-mercaptoethanol	Gibco	31350-010	50 µM

Expansion medium			
N2B27 medium			
EGF	Sigma	E9644	10 ng/mL
FGF	PeproTech	100-18B	10 ng/mL

Differentiation medium			
N2B27 medium			
BDNF	PeproTech	450–02	20 ng/mL
GDNF	PeproTech	450–10	10 ng/mL
Ascorbic acid	Sigma	A4544	100 µM
db-cAMP	Sigma	D0627	10 µM

### OrganoPlate NVU culture

The OrganoPlate 3-lane platform (4004-400B, MIMETAS) was used for all experiments. Channel dimensions are 400 µm × 220 µm (w × h) and phaseguides had dimensions of 100 µm × 55 µm (w × h). Rat-tail collagen-I gel was prepared as previously described [51, 52] and dispensed in the middle lane of OrganoPlate 3-lane tissue chips by adding 2 µL to the gel inlet. After 15 min of gelation at 37 °C, a Matrigel-GFR coating (80–100 µg/mL in cold PBS) was added to the bottom channel of each chip by pipetting 40 µL into the inlet wells. The OrganoPlate was incubated at 37 °C overnight. Next, astrocytes and three-week pre-differentiated neurons were thawed, pelleted, and resuspended in differentiation medium at a density of 15,000 cells/µL, in a 1:4 ratio. 1–2 µL of astrocyte-neuron cell suspension were seeded in the bottom channel of each chip using passive pumping technique [53]. In short, Matrigel-GFR coating was aspirated from the bottom inlet of each chip and replaced with 50 µL of differentiation medium, after which the cell suspension was seeded on the connecting outlet, causing the cells to get drawn into the channel. The OrganoPlate was placed static at 37 °C for 1 h to allow cell attachment, after which medium was aspirated from the bottom inlet of the chips. Differentiation medium was then added to the top inlet and outlet wells (50 µL each) of each chip and the OrganoPlate was placed on the OrganoFlow perfusion rocker (MIMETAS, OFPR-L, 7° inclination, 8-min interval). Medium was changed twice a week by aspirating medium from the inlet and outlet wells of the top channel of each chip and replacing it with fresh differentiation medium. After 7 days, HBMECs (10,000 cells/µL) were seeded to the top channel of each chip using the passive pumping technique [53]. The OrganoPlate was incubated on the side for 3 h in the incubator to allow the HBMECs to sediment against the collagen-I gel and attach. After 3 h, all medium was aspirated from the chips and fresh medium was added. Endothelial cell medium (OrganoMedium, HBMECBM, MIMETAS) was added to the top channel (50 µL in inlet, 50 µL in outlet) and neuronal differentiation medium (see Table 1) was added to the bottom channel (50 µL in inlet, 50 µL in outlet) of each chip. The OrganoPlate was placed back on the OrganoFlow perfusion rocker and culture (37 °C, atmospheric (20%) O_2_, 5% CO_2_) was continued. Medium changes were performed 2–3 times a week. Assays were performed on day 14–15. A schematic representation of NVU culture in the OrganoPlate is shown in Additional file 1.

### Modeling stroke

Stroke was modeled using a three-fold approach for a duration of 16 h, starting on day 14 of culture. Hypoglycemic conditions were modeled by replacing the culture media on both sides of the chips with glucose-free formulations. OrganoMedium HBMECBM-GF (MIMETAS) was added to the top channel (50 µL in inlet, 50 µL in outlet) of each chip. Neuronal differentiation was prepared as usual (see Table 1), but with Neurobasal-A medium (A2477501, ThermoFisher) instead of glucose-containing Neurobasal medium. Hypoxic conditions were modeled using 10 µM antimycin-A (A8674, Sigma), an inhibitor of complex III of the electron transport chain. Disrupted perfusion was modeled by removing the OrganoPlate from the rocker platform and placing it static in the incubator. Each approach was tested individually and in combination.

### Immunocytochemistry

Cultures in the OrganoPlate were fixed with 3.7% formaldehyde (252549, Sigma) or 100% methanol (− 20 °C, 494437, Sigma) and immunostained as previously described [51]. In short, cells were permeabilized using a Triton X-100 solution for 10 min and blocked using a buffer containing FBS, bovine serum albumin, and Tween-20 for 45 min. Primary antibody was incubated for 1–2 h or overnight, after which secondary antibody was incubated for 1 h. For negative controls, incubation with primary antibody was omitted and only incubation with secondary antibody was performed. An overview of the antibodies used can be found in Table 2. Nuclei were stained using Hoechst (H3570, ThermoFisher) and cells were imaged with ImageXpress Micro XLS and Micro XLS-C HCI Systems (Molecular Devices).

**Table 2:** Antibodies used for immunofluorescent staining

Primary antibodies					
Antibody	Supplier	Cat. no	Dilution	Fixation	Immunogen
VE-cadherin	Abcam	ab33168	1:1000	Formaldehyde	Synthetic peptide corresponding to Human VE Cadherin aa 750 to the C-terminus conjugated to keyhole limpet haemocyanin
PECAM-1	Dako	M0823	1:20	Formaldehyde	The epitope recognized was found to be within the extracellular domain 1
ZO-1	ThermoFisher	339100	1:100	Formaldehyde	Human recombinant ZO-1 fusion protein encompassing amino acids 334–634
Claudin-5	ThermoFisher	35-2500	1:70	Methanol	Synthetic peptide derived from the mouse Claudin-5 protein
S100β	Abcam	Ab52642	1:100	Formaldehyde	Synthetic peptide within Human S100 beta aa 50 to the C-terminus (C terminal). The exact sequence is proprietary
GFAP	Abcam	Ab4674	1:1000	Formaldehyde	Recombinant full length protein corresponding to Human GFAP. Isotype 1 expressed in and purified from *E. coli*
B3TUBB	Novus Biologicals	NB100-1612	1:500	Formaldehyde	Chickens were immunized with synthetic peptides that corresponded to different regions of beta-III Tubulin, but are shared between the human (NP_AAL28094, NCBI) and rat (AAM28438, NCBI) protein sequences
B3TUBB	Abcam	Ab78078	1:100	Formaldehyde	Synthetic peptide corresponding to Rat beta III Tubulin aa 400–500

Secondary antibodies				
Antibody	Supplier	Cat. no	Dilution	Immunogen
Goat-anti-rabbit Alexa 488+	ThermoFisher	A11008	1:250	Gamma Immunoglobins Heavy and Light chains
Goat-anti-rabbit Alexa 555	ThermoFisher	A21428	1:250	Gamma Immunoglobins Heavy and Light chains
Goat-anti-mouse Alexa 488	ThermoFisher	A11001	1:250	Gamma Immunoglobins Heavy and Light chains
Goat-anti-mouse Alexa 555+	ThermoFisher	A21422	1:250	Gamma Immunoglobins Heavy and Light chains
Goat-anti-chicken Alexa 647	ThermoFisher	A21449	1:500	Gamma Immunoglobins Heavy and Light chains
Donkey-anti-mouse Alexa 647	ThermoFisher	A31571	1:250	Gamma Immunoglobins Heavy and Light chains

### Calcium imaging

Cells were incubated with Cal-520 (20 µM, ab171868, Abcam) and 0.4% Pluronic F-127 (P6866, Invitrogen) in serum-free medium for 60 min at 37 °C on the rocker platform, followed by 30 min at RT. Fluorescent images were acquired at 0.5 Hz, 4×, widefield setting, 20% FITC-intensity using the Micro XLS-C HCI System (Molecular Devices). Calcium recordings were corrected for photo-bleaching using a bleach correction plugin in Fiji [54]. For quantification, regions of interest were selected and fluorescent signal over time was plotted for selected individual neurons using Fiji multi measure functionality. Graphs were plotted using GraphPad Prism 8 (GraphPad Software).

### Transendothelial electrical resistance

Transendothelial electrical resistance (TEER) was measured at different time points using an automated multichannel impedance spectrometer designed for use with the OrganoPlate (OrganoTEER, MI-OT-1, MIMETAS). Before the baseline measurement, 50 µL HBSS was added to the middle inlets and outlets of each OrganoPlate chip. Medium solutions containing staurosporine (S4400, Sigma) or vehicle (0.001% DMSO) were dispensed into a multiwell plate. The OrganoPlate, medium solutions and TEER equipment were equilibrated in the incubator (37 °C, 5% CO_2_) for at least 30 min prior to the start of the experiment. The OrganoPlate was placed in the OrganoTEER, allowing electrode pairs to be inserted into all inlet and outlet wells. Point impedance measurements were performed in the incubator (37 °C, 5% CO_2_) by frequency sweep from 1000 Hz to 1 MHz (100 points; precision 0.5). A baseline measurement was performed first (1 timepoint only). Medium was then aspirated and replaced with vehicle or staurosporine-containing medium. Time-lapse measurements were then performed every 16 min for a total of 23 h and 45 min (91 timepoints). Data were analyzed using the OrganoTEER software, which automatically extracts the TEER contribution (in Ohm) from the measured spectra and normalizes it to Ohm*cm^2^ by multiplying by the microvessel-ECM interface (estimated at 0.0057 cm^2^).

### Permeability assay

Barrier permeability assays were performed as previously described [52]. In short, chips were wetted with culture medium to ensure proper flow profiles and medium was aspirated from the chips. 20 µL of medium without fluorescent compound was added to the basal side of the chips (the gel inlet and outlet well and the bottom inlet and outlet well). Medium containing a fluorescent dye (10 µg/mL sodium fluorescein, F6377, Sigma; or 0.1 mg/mL 20 kDa FITC-dextran, FD20S, Sigma) was perfused through the lumen of the endothelial microvessel in the top channel (40 µL in inlet well, 30 µL in outlet well). Images were acquired from each chip every 2 min for a duration of 10 min using an ImageXpress XLS Micro HCI System (Molecular Devices). The leakage score was calculated as follows: [fluorescent signal in ECM gel channel]/[fluorescent signal in lumen of endothelial vessel]. Apparent permeability (P_app_) of each HBMEC vessel was calculated as previously described [55]. Graphs were plotted using GraphPad Prism 8 (GraphPad Software).

### Transporter expression

RNA was collected from proliferating 2D HBMEC cultures (1 × T75 flask, cultured in MV2 medium) or from the HBMEC fraction of NVU on-a-chip cultures by lysation with RLT buffer (79216, Qiagen). For NVU on-a-chip cultures, lysates from 5 chips were pooled into one sample before proceeding to RNA isolation. RNA isolation was performed using an RNeasy Micro kit (74004, Qiagen) according to manufacturer’s instructions. RNA yield and purity were measured using the NanoDrop OneC Microvolume UV–Vis Spectrophotometer (ND-ONE-W, ThermoFisher). Complementary DNA was synthesized using M-MLV reverse transcriptase (28025013, ThermoFisher) according to manufacturer’s instructions. The following primers were used in this study: Glut-1 (forward: 5′-AACTCTTCAGCCAGGGTCCAC-3′, reverse: 5′-CACAGTGAAGATGATGAAGAC-3′), P-gp (forward: 5′-GGCACCAACATGGACAACC-3′, reverse: 5′-GTATTCCTGGGACACGCAGT-3′), BCRP1 (forward: 5′-AGATGGGTTTCCAAGCGTTCAT, reverse: 5′-CCAGTCCCAGTACGACTGTGACA-3′), MRP1 (forward: 5′-GCCGAAGGAGAGATCATC-3′, reverse: 5′-AACCCGAAAACAAAACAGG), TfR (forward: 5′-CTGCTATGGGACTATTGCTGTG-3′, reverse: CGACAACTTTCTCTTCAGGTC), and ACTB (forward: 5′-CTCTTCCAGCCTTCCTTCCT-3′, reverse: 5′-AGCACTGTGTTGGCGTACAG-3′). Quantitative PCR was performed using FastStart Essential DNA Green Master (06402712001, Roche) on the LightCycler® 96 Instrument (05815916001, Roche), performing each measurement in triplicate (technical replicates). The data was analyzed using the corresponding software according to the ΔΔCq method [56]. In brief, (1) ΔCq, (2) ΔΔCq, and (3) fold change were calculated in Microsoft Excel using the following equations: 1) ΔCq = Cq gene of interest – Cq endogenous control; 2) ΔΔCq = (Cq gene of interest – Cq endogenous control)_sample A_ – (Cq gene of interest – Cq endogenous control)_sample B_; and 3) Fold change = 2-ΔΔCq. Target genes were normalized to the endogenous control beta-actin. Fold changes for NVU on-a-chip cultures were calculated relative to control cultures (2D HBMEC). Graphs were plotted using GraphPad 8 (GraphPad Prism Software).

### P-glycoprotein functionality

P-glycoprotein (P-gp) assays were performed as previously described [55]. In short, calcein-AM (C3099, ThermoFisher), a substrate of P-gp, was perfused through the lumen of the model in presence or absence of P-gp inhibitor cyclosporin-A (30042, Sigma) or zosuquidar (SML1044, Sigma) for 60 min. Next, the chips were washed with cold Opti-HBSS buffer (1:3 mix of Opti-MEM, 31985062, Thermo Fisher and HBSS, H6648, Sigma) and perfused with Hoechst (H3570, ThermoFisher) to stain the nuclei. Z-stacks were acquired using the ImageXpress® Micro Confocal High Content Imaging System (Molecular Devices) and green-fluorescent calcein signal was normalized to Hoechst cell count to quantify the intracellular calcein level in conditions with and without P-gp inhibitor. Graphs were plotted using GraphPad Prism 8 (GraphPad Software).

### Mitochondrial membrane potential assay

Tetramethylrhodamine, methyl ester (TMRM, T668, ThermoFisher) was used to determine the mitochondrial membrane potential according to manufacturer’s instructions. Medium was aspirated from all inlets and outlets and 50 µL of 250 nM TMRM staining solution was added to the top and bottom inlet and outlet wells of the chips. The plate was incubated at 37 °C on the rocker platform for 30 min followed by washing with HBSS (55037C, Sigma). Nuclei were stained using Hoechst (H3570, ThermoFisher) and z-stacks were acquired using the ImageXpress® Micro Confocal HCI System (Molecular Devices). SUM projections were loaded into Fiji and a region of interest was selected to extract a mean intensity value from each chip. Background signal was subtracted by deducting the mean intensity from cell-free control chips from each chip. Graphs were plotted using using GraphPad Prism 8 (GraphPad Software).

### ATP assay

A CellTiter-Glo® 3D cell viability assay (G9681, Promega) was used according to manufacturer’s instructions. ATP standard curve solutions (0.016-0.08-0.4-2-10-20 µM) were prepared using ATP disodium salt (A7699, Sigma) in HBSS (55037C, Sigma). CellTiter-Glo® 3D ready-to-use reagent was mixed in a 1:1 ratio with HBSS and added to the OrganoPlate chips (50 µL in top and bottom inlets and outlets) for 15 min incubation at 37 °C on the rocker. The ATP standard curve solutions and the lysates from the OrganoPlate cultures were transferred to white, flat bottom 384-well plates (262360, ThermoFisher). Luminescence was measured in duplo for each sample using a multiwell plate reader (Fluoroskan Ascent®, ThermoFisher). ATP concentrations of the OrganoPlate samples were interpolated from the luminescent values following calibration curve fitting in GraphPad Prism 8 (GraphPad Software). Graphs were plotted using the same software.

### Statistical analyses

Data was analyzed using GraphPad Prism, version 8. Gaussian distribution was assessed using the Shapiro–Wilk normality test. In case the assumptions were not violated, one-way ANOVAs were performed. In case of normally distributed data in which the assumption of equality of variances was violated (Figs. 4d, 5a–d), the Brown-Forsythe and Welch test was performed with a Dunnett T3 multiple comparisons test. When normal distribution could not be confirmed (Fig. 4b, left panel graph), the nonparametric Kruskall-Willis test with Dunn’s multiple comparisons test was performed. A 2-way ANOVA with Sidak’s multiple comparison test was used to compare vehicle control data of the two graphs shown in Fig. 4b (with medium composition as variable 1 and presence or lack of perfusion as variable 2). For multiple comparisons tests, all groups were compared to the vehicle control group only. Statistical significance was indicated by one or more asterisks. *P < 0.05, **P < 0.01, ***P < 0.001, or ****P < 0.0001.

## Results

### Perfused 3D NVU on-a-chip shows a vessel of brain endothelial cells in co-culture with astrocytes and neurons

**Fig. 1:**
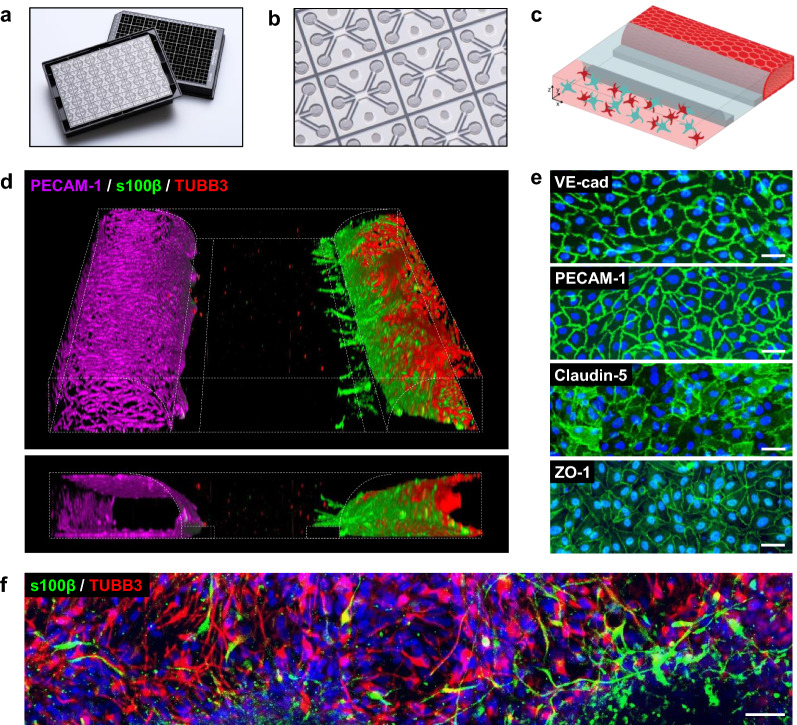
Brain endothelial cells, astrocytes, and neurons in a 3D NVU on-a-chip. **a** Picture of the OrganoPlate 3-lane culture platform, comprising 40 tissue culture chips. **b** Picture of the bottom of the OrganoPlate, showing several 3-lane chips. **c** 3D artist impression of the NVU on-a-chip model. **d** 3D reconstruction of the human NVU model showing a vessel of brain endothelial cells (PECAM-1, magenta) grown against an extracellular matrix gel, in co-culture with networks of astrocytes (s100β, green) and neurons (TUBB3, red). **e** Single-plane images of the brain endothelial cells that make up the endothelial vessel in the top lane of the chips, expressing adherens junction markers VE-cadherin and PECAM-1, and tight junction markers claudin-5 and ZO-1. **f** Astrocytes (s100β, green) and neurons (TUBB3, red) are present in the bottom lane of the chips and form networks. All images were acquired from 14-day old cultures. Panels show representative images of n = 2–3 chips minimum. Scale bars are 50 µm

The OrganoPlate 3-lane allows parallel culture of 40 miniaturized tissues in microfluidic chips (Fig. 1a, b) [50–52]. In each chip, a 3D human NVU triculture model was grown under medium perfusion (Fig. 1c, Additional file 1). The NVU cultures were characterized by immunostaining. Figure 1d shows a 3D reconstruction of a representative NVU on-a-chip culture. The culture comprised a vessel of brain endothelial cells, grown against a collagen-I gel, in co-culture with neurons and astrocytes. Cultures remained viable for a minimum of two weeks (Additional file 2).

The vessel of primary brain endothelial cells showed expression of adherens junction proteins vascular endothelial cadherin (VE-cadherin) and platelet endothelial cell adhesion molecule 1 (PECAM-1) as well as tight junction proteins claudin-5 and zona occludens 1 (ZO-1) (Fig. 1e). The presence of these markers and their localization at the cell–cell contacts is indicative of barrier formation [1, 57]. Located on the opposite side of the collagen-I gel were networks of iPSC-derived neurons and astrocytes, positive for neuronal marker beta-III-tubulin (TUBB3) and astrocytic marker astrocytic marker S100 calcium-binding protein B (s100β), respectively (Fig. 1f).

To ensure neuronal functionality, electrophysiological activity was detected by means of calcium imaging [58], confirming spontaneous neuronal firing in the NVU cultures (Additional file 3).

### The NVU on-a-chip model shows tight barrier function and allows study of compound-induced barrier disruption

After observing expression of adherens- and tight junction proteins in the NVU on-a-chip cultures, we investigated barrier formation of the endothelial vessel at a functional level, at baseline and in response to staurosporine, an anticancer drug that disrupts BBB integrity and induces apoptosis [59, 60]. NVU on-a-chip cultures were exposed to two concentrations of staurosporine for a duration of 24 h. To assess barrier integrity, TEER was measured over time during the exposure.

**Fig. 2:**
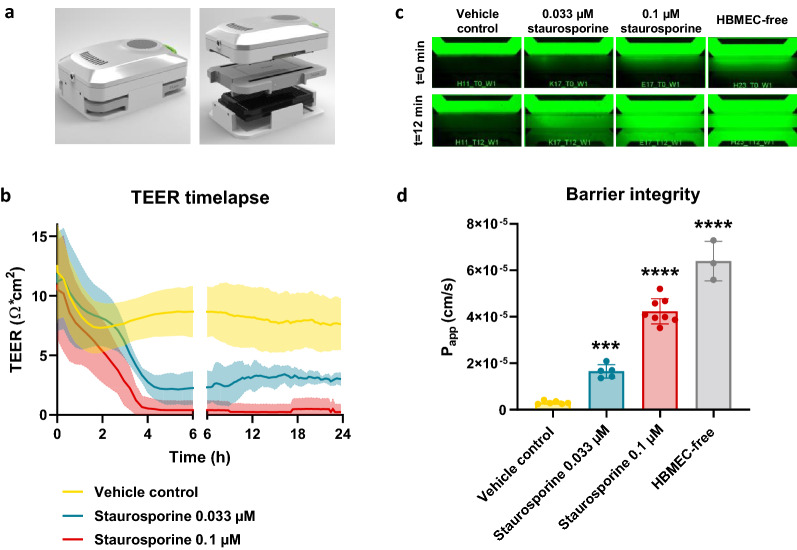
NVUs on-chips are leak-tight for small molecule sodium fluorescein and allow study of compound-induced barrier disruption. **a** The OrganoTEER device is employed to assess transendothelial electrical resistance (TEER) in NVU on-a-chip cultures grown in the OrganoPlate 3-lane. **b** Timelapse TEER measurements of NVU on-a-chip cultures exposed to staurosporine (0.033 or 0.1 µM) or vehicle control for 24 h (day 14–15 of culture). Graph shows mean ± standard deviation in the form of a shaded error envelope, n = 5–8 chips. **c** After 24 h exposure to staurosporine or vehicle control, the cultures’ barrier integrity was assessed by addition of sodium fluorescein (0.45 nm) to the lumen of the cultures. Images were acquired every 2 min for a duration of 12 min. Figure shows representative images acquired at the start (t = 0 min) and end (t = 12 min) of the assay. **d** Quantification of apparent permeability (P_app_) of sodium fluorescein in NVU on-a-chip cultures exposed to staurosporine or vehicle control and compared to an HBMEC-free (endothelial barrier-free) culture. Graph shows mean ± standard deviation, n = 3–8 chips. Statistical analysis was performed using one-way ANOVA; *P < 0.05, **P < 0.01, ***P < 0.001, ****P < 0.0001

The measurements showed a concentration-dependent decrease in TEER in response to staurosporine (Fig. 2a, b). The lower concentration of 0.033 µM resulted in a near-linear decrease in TEER during the first 4 h of exposure before reaching a plateau that showed a 66% reduction in TEER compared to vehicle control. The higher concentration of 0.1 µM staurosporine results in near-zero TEER values after 4 h of exposure, which remained unchanged for the remainder of the experiment.

Following the 24 h exposure to staurosporine, the barrier integrity of each chip was also assessed using a fluorescent assay. Small molecule sodium fluorescein (0.45 nm radius [61]) was added to the lumen of each chip and its leakage into the adjacent gel lane was monitored over time by timelapse imaging and quantification. In line with the TEER measurements, a concentration-dependent decrease in barrier function was observed (Fig. 2c, d), with untreated chips proving leak-tight for sodium fluorescein (P_app_ of 3.1 × 10^–6^ cm/s) and treated chips showing leakage (P_app_ of 4.2 × 10^–5^ cm/s and 1.6 × 10^–5^ cm/s for 0.033 µM and 1 µM staurosporine, respectively).

### The NVU on-a-chip expresses relevant BBB transporters

**Fig. 3:**
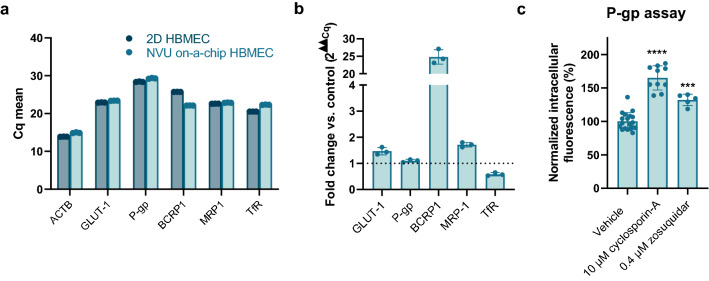
NVU on-a-chip expresses relevant BBB transporters and exhibits P-gp functionality. **a** RNA was isolated from 2D HBMEC cultures or HBMECs cultured in the NVU on-a-chip model. Graph shows mean Cq values for transporters GLUT-1, P-gp, BCRP1, MPR1, and TfR compared to housekeeping gene actin beta (ACTB). Each sample was measured in triplicate, with individual data points representing technical replicates of one biological replicate sample. **b** Expression of GLUT-1, P-gp, BCRP1, MRP1, and TfR in HBMECs cultured in the NVU on-a-chip model expressed as fold change compared to 2D cultured HBMECs. **c** Calcein-AM, a substrate of efflux transporter P-gp, was perfused through the lumen of the endothelial vessel and was taken up by the cells and converted to fluorescent calcein. P-gp’s ability to efflux calcein out of the cell was inhibited using cyclosporin-A or zosuquidar, resulting in an increase in intracellular fluorescence. n = 5–19 chips. Graphs show mean ± standard deviation. Statistical analysis was performed using one-way ANOVA; *P < 0.05, **P < 0.01, ***P < 0.001, ****P < 0.0001

After confirming the barrier function of the NVU on-a-chip model, we assessed expression of relevant transporters. Expression of influx transporter GLUT-1 and efflux transporters P-gp, breast cancer resistance protein 1 (BCRP1), and multidrug resistance protein 1 (MRP1) were confirmed at the RNA level. In addition, we confirmed expression of the transferrin receptor (TfR), which is of interest for drug delivery of biologicals into the brain (Fig. 3a). P-gp expression was similar in NVU on-a-chip cultures compared to HBMECs cultured in 2D. A small upregulation was observed for GLUT-1 and MRP1, while a small downregulation was observed for TfR. Notably, a 24-fold increase was found for BCRP1 (Fig. 3b).

A functional assay was employed to confirm activity of one of these transporters, namely P-gp, an important member of the ATP-binding cassette family and involved in drug efflux from the brain. The lumen of NVU on-a-chip cultures was perfused with calcein-AM, a fluorescent substrate of P-gp [62–64]. Inhibition of P-gp with first-generation inhibitor cyclosporin-A [65, 66] (10 µM, 60 min) or the more selective third-generation inhibitor zosuquidar [66] (0.4 µM, 60 min) resulted in a significant increase in intracellular fluorescence (65% and 32%, respectively), indicative of inhibited efflux of the fluorescent substrate out of the cells (Fig. 3c).

### Mimicking ischemic stroke induces barrier leakage, reduced mitochondrial membrane potential, and lowered ATP

**Fig. 4:**
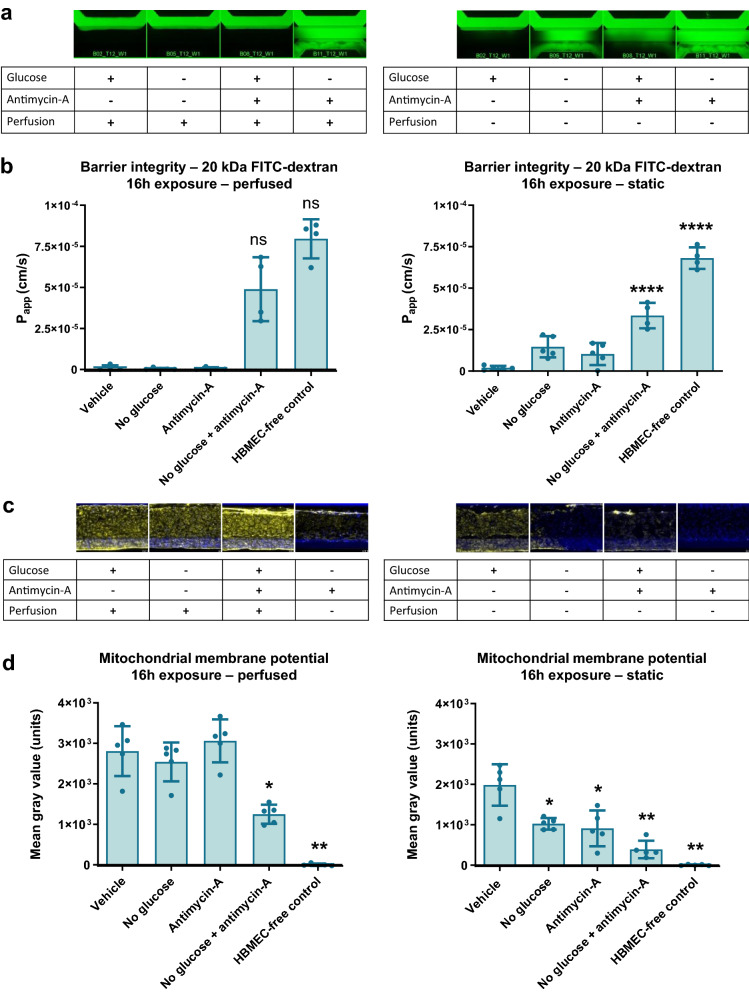
Ischemic conditions cause a disruption in barrier function and reduced mitochondrial potential. The NVU model was exposed to conditions that mimic stroke for 16 h (day 14–15 of culture) by omitting glucose, addition of antimycin-A to model hypoxia, and discontinuation of flow. **a** Fluorescent images show representative leakage for each condition 10 min after addition of 20 kDa FITC-dextran. **b** Quantification of apparent permeability (P_app_) of 20 kDa FITC-dextran in all conditions. Graphs show mean ± standard deviation, n = 4–5 chips. Statistical analysis was performed using Kruskal–Wallis tests for left panel graph (in which normal distribution was not confirmed) and one-way ANOVA for right panel graph; ns (not significant), *P < 0.05, **P < 0.01, ***P < 0.001. **c** Mitochondrial potential was assessed using a fluorescent TMRM assay in which a high fluorescent signal corresponds with healthy mitochondrial function. Fluorescent images show representative TMRM signal for each condition. **d** Quantification of TMRM signal in all conditions. Graphs show mean ± standard deviation, n = 4–5 chips. Statistical analysis was performed using Brown-Forsythe and Welch tests; *P < 0.05, **P < 0.01

Next, the NVU on-a-chip cultures were used to investigate the effects of ischemic stroke on NVU function. Stroke was mimicked using a threefold approach: (1) glucose depletion was modeled using glucose-free medium, (2) hypoxia was mimicked using antimycin-A [22, 26], an inhibitor of complex III of the electron transport chain, and (3) halted perfusion was mimicked by removing the OrganoPlate from the rocker platform and placing it static. Each approach was assessed separately and in combination with the other approaches and compared to control chips. Results showed that when perfusion is continued, no-glucose alone or antimycin-A alone do not alter NVU barrier integrity, but the combination of no-glucose and antimycin-A induces a marked disruption of the endothelial barrier, causing leakage of 20 kDa FITC-dextran out of the models’ lumen (Fig. 4a, b, left panels; P_app_ of 4.9 × 10^–5^ cm/s versus 9.3 × 10^–7^ cm/s for vehicle control). In contrast, in absence of perfusion, no-glucose alone or antimycin-A alone both did cause a decrease in barrier integrity compared to control. A combination of no-glucose and antimycin-A in absence of perfusion again showed a further disruption of barrier integrity (Fig. 4a, b, right panels; P_app_ of 3.3 × 10^–5^ cm/s versus 2.0 × 10^–6^ cm/s for vehicle control).

Similar effects were observed when studying mitochondrial function using a fluorescent mitochondrial membrane potential assay. Under perfusion, no-glucose alone or antimycin-A alone did not significantly alter mitochondrial membrane potential, but the combination of no-glucose and antimycin-A caused a strong decrease (Fig. 4c, d, left panels; 55.6% reduction in fluorescent intensity compared to vehicle control). In absences of perfusion, mitochondrial membrane potential was drastically reduced in all conditions compared to perfused chips, with also control chips (with glucose, without antimycin-A) showing significantly lowered mitochondrial membrane potential (Fig. 4c, d right panels; 29.3% reduction compared to perfused vehicle control, P = 0.0079). In line with results from the barrier integrity assay, mitochondrial membrane potential was also affected in no-glucose only and antimycin-A only conditions if perfusion was stopped. The strongest reduction was again observed when both approaches were combined (80.5% reduction compared to static vehicle control; 86.2% compared to perfused vehicle control).

In stroke, the disrupted blood flow to the brain leads to hypoxic and hypoglycemic conditions and hence a reduction in ATP, the molecule that supplies energy for processes in living cells. Using a CellTiter-GLO assay, we assessed ATP levels in the apical (blood) and basal (brain) side of the NVU on-a-chip cultures after applying stroke conditions.

**Fig. 5:**
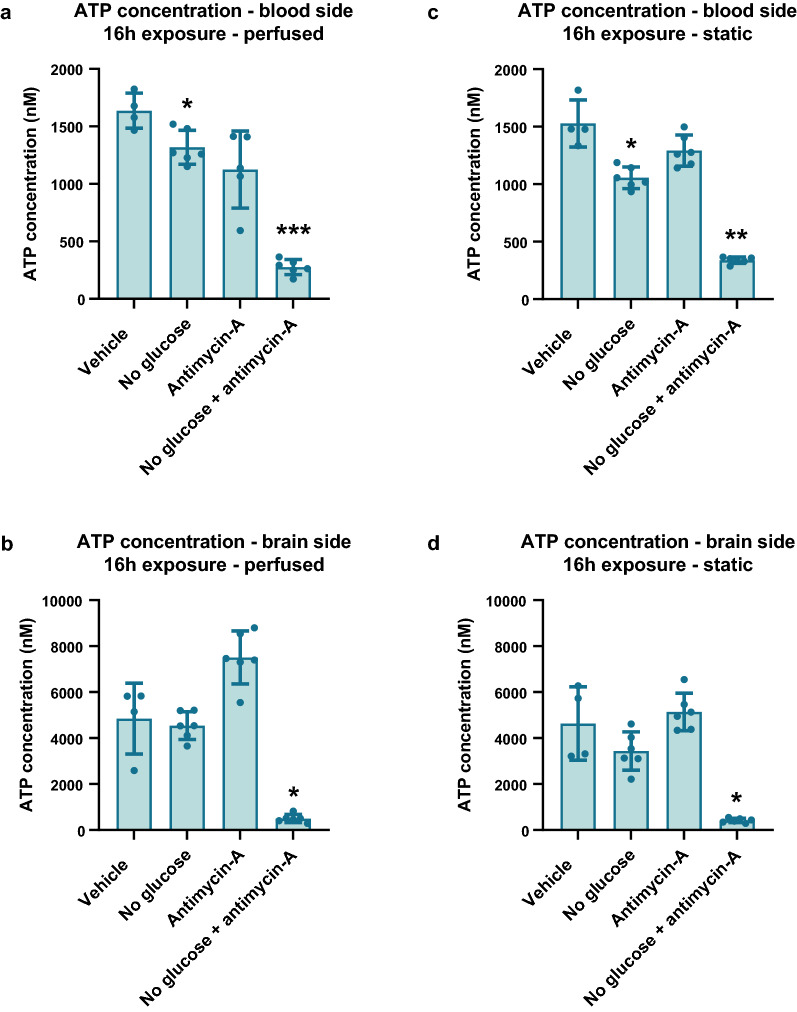
Ischemic conditions cause a reduction in ATP in the neurovascular unit model. The NVU model was exposed to conditions that mimic stroke for 16 h (day 14–15 of culture) by omitting glucose, addition of antimycin-A to model hypoxia, and discontinuation of flow. **a** ATP concentration on blood side of NVU on-a-chip in condition with continued perfusion. **b** ATP concentration on brain side of NVU on-a-chip in condition with continued perfusion. **c** ATP concentration on blood side of NVU on-a-chip in condition with halted perfusion. **d** ATP concentration on brain side of NVU on-a-chip in condition with halted perfusion. Graphs show mean ± standard deviation, n = 4–5 chips. Statistical analysis was performed using Brown-Forsythe and Welch tests; *P < 0.05, **P < 0.01, ***P < 0.001

Under perfused conditions, no-glucose alone or antimycin-A alone resulted in a reduction in ATP levels on the blood side of the chips compared to vehicle control (19.4% and 31.3% reduction, respectively) (Fig. 5a). The combination of glucose removal and presence of antimycin-A resulted in a strong decrease in ATP levels (83% reduction). Samples taken from the brain side of the perfused chips showed higher baseline ATP levels than on the blood side, explained by the larger number of cells present in that compartment. On the brain side of the perfused chips, no-glucose alone did not affect ATP levels (Fig. 5b). Addition of antimycin-A showed a trend of increased ATP, although not significant. Like the blood side, the brain side of the perfused chips showed a strong reduction in ATP when no-glucose and antimycin-A were combined (89.7%).

When perfusion was halted, the blood side of the chip showed similar findings as when perfusion was continued, with combination of no-glucose and antimycin-A showing a strong decrease in ATP concentrations (77.8% reduction) (Fig. 5c). The brain side of the chips again showed higher baseline ATP levels due to a larger number of cells present. In samples taken from the brain side, no-glucose alone or antimycin-A alone did not significantly alter ATP concentrations (Fig. 5d). A combination of the two, however, resulted in strongly decreased ATP (91.1%).

## Discussion

We established a triculture NVU on-a-chip model that accounts for many key features of the NVU without the need for cumbersome procedures or long culture times. The NVU on-a-chip model consists of a vessel of primary brain endothelial cells in co-culture with iPSC-derived astrocytes and neurons. The model presents with tight in vivo-like barrier function as observed by the retention of the small molecule sodium fluorescein*.* Exposure of the NVU on-a-chip model to a known disruptive compound decreased the TEER of the barrier and increased its permeability to sodium fluorescein. This finding indicates the model’s use in assessing BBB-disrupting compounds and potential restorative therapies. In addition to tight barrier function, the NVU on-a-chip cultures also demonstrated spontaneously active neurons and expression of relevant endothelial transporters.

The NVU on-a-chip model presented with an average TEER of 12.6 Ω  × cm^2^ at baseline. Although TEER measurements can in theory be compared between different culture setups and systems, practice shows otherwise [67–69]. Highly conflicting TEER values have been reported by different studies even when the same cells and culture setups were used. When different culture systems are employed, such as Transwell and microfluidic systems, the discrepancies are even larger. The discrepancies result from a combination of different factors that influence TEER values, including thickness and pore size of membranes, electrode type and position, electrical and mathematical approaches, temperature, surface area and shape, and current line distribution [70]. For this reason, we recommend that TEER values are compared only within a study rather than between studies. Additional permeability studies using fluorescent molecules and imaging-based readouts can aid in further characterization of a model’s barrier function. In this study, we observed a concentration-dependent loss of barrier function over time using the BBB-disrupting anticancer drug staurosporine [59, 60]. While exposure to 0.033 µM staurosporine resulted in a 66% reduction in TEER compared to vehicle control, exposure to 0.1 µM staurosporine resulted in a near complete loss of TEER. These findings were supported by a concentration-dependent increase in leakage of fluorescent molecule sodium fluorescein. These findings indicate that TEER measurements in our NVU on-a-chip system can be used to assess disruption of the NVU’s barrier.

In addition to TEER, we assessed barrier function of NVU on-a-chip cultures using sodium fluorescein, a commonly used small molecule dye for studying BBB permeability [71–74]. Hawkins et al. reported that sodium fluorescein is subject to transport by organic anion transporter 3 (Oat3) and MRP2 in rats and therefore may result in an overestimation of a culture’s barrier properties [75]. Although sodium fluorescein presents with this disadvantage, it presents with several other highly favorable characteristics [74], including its inability to accumulate inside cells [76]. We have assessed both sodium fluorescein and lucifer yellow, a dye that has not been reported to be substrate to transport, in HBMEC monocultures and found that the cellular barriers retained both dyes (data not shown). Moreover, the permeability for sodium fluorescein in our NVU on-a-chip cultures (P_app_ of 3.08 × 10^–6^ cm/s) falls within the range of the molecule’s in vivo permeability reported for rat brain microvessels (P_app_ of 0.11 × 10^–6^ [75] to 2.71 ± 0.76 × 10^–6^ cm/s) [77]), indicating tight barrier formation. We do recommend that like TEER, P_app_ values are also compared within a study rather than between studies, as they are also subject to large discrepancies in reported values due to biological as well as technical and analytical parameters [78].

Expression of BBB transporters was confirmed at the RNA level for glucose influx transporter GLUT-1, efflux transporters P-gp, BCRP, and MRP1, as well as for TfR, an important transporter for receptor mediated transcytosis. In the presence of P-gp inhibitors cyclosporin-A [65, 66] or zosuquidar [66], accumulation of fluorescent P-gp substrate calcein was observed, indicative of functional P-gp activity [62–64]. We have not performed functional assessment of other relevant transporters. Future work may include functional assessment of BCRP1, which was shown to be upregulated in the NVU on-a-chip model compared to 2D, and GLUT-1, which is reported to become upregulated following ischemic stroke [79]. Lastly, investigation of the sodium-dependent glucose transporter (SGLT) may be of interest, as some have reported a combined role for GLUT-1 and SGLT in ischemic stroke [80].

Following previous work with cell lines [51], the NVU on-a-chip presented here employs primary brain endothelial cells. Potential concerns with the use of primary brain endothelial cells include dedifferentiation and loss of certain characteristic features after removal from their in vivo environment [81, 82]. In addition, the use of primary cells is subject to donor variation. For this reason, the use of iPSC-derived brain endothelial cells (iBECs) has gained much attention over the last decade. However, recent studies acknowledge that the current protocols for iBEC generation often result in suboptimal cellular phenotypes [46, 83, 84], showing a predominantly epithelial phenotype and a lack of active transport across the cells. Our experience with iBECs is in line with these reports (data not shown). For this reason, we employed primary human brain endothelial cells in our NVU on-a-chip model. The resulting endothelial vessel shows a relevant phenotype, including expression of relevant BBB transporters and tight barrier formation. For consistency, endothelial cells from only one donor were used in this study. However, we have worked with three different donors without finding obvious donor to donor differences (data not shown), indicating that donor variation does not necessarily pose insurmountable issues. Lastly, the use of primary brain endothelial cells rather than iBECs also allows for strongly reduced culture times. As the field continues to improve iBEC differentiation protocols, the replacement of primary brain endothelial cells by iPSC-derived ones in our NVU on-a-chip model may be possible in the near feature when for example donor matched models are desired for personalized medicine applications.

The OrganoPlate platform allows flexible tissue model design [85]. In addition to the cell types present in the model described here, one could easily add pericytes, which play a major role in healthy NVU functioning [3, 51]. The model can also be expanded to include microglia, the resident macrophages of the brain [86, 87]. Additionally, the role of circulating immune cells may be investigated. Impaired BBB function and inflammation is observed in many acute and chronic neurological diseases and results in the entrance of immune cells from the systemic circulation into the brain [8, 88, 89]. After entering the brain these immune cells further exacerbate BBB disruption, either directly—via release of inflammatory factors such as cytokines, free radicals and matrix metalloproteinases—or indirectly, via activation of other constituents of the NVU, such as astrocytes or microglia [90–92]. The expression of endothelial cell adhesion molecules (CAMs) such as intercellular CAM-1 (ICAM-1) can be studied in our NVU on-a-chip model at baseline and after mimicking stroke or other neurological disorders. Immune cells can be perfused through the lumen of the endothelial vessel in the OrganoPlate and immune cell adhesion to the vascular wall can be quantified, as reported by Poussin et al. [93]. Subsequent extravasation and migration towards the brain side of the chip may be studied using an approach similar to the one reported by Gjorevski et al. [94] or De Haan et al. [95]. Furthermore, samples can be taken from apical and basal compartments and cytokine contents can be analyzed, as shown in a study by Gijzen et al. [85].

This study modeled ischemic stroke using a threefold approach that combines hypoglycemia, chemical hypoxia, and halted perfusion. When perfused was continued, omission of glucose alone showed limited effects on NVU barrier function, endothelial mitochondrial membrane potential, or ATP levels on blood- and brain side. This may be explained by a compensatory mechanism, such as a switch to cellular respiration mechanisms that don’t rely on the presence of glucose. Upon low glucose levels, endothelial cells have shown to increase fatty acid oxidation, also known as β-oxidation, for energy production [96, 97]. When perfusion is halted, delivery of new fatty acids is hampered, possibly explaining why the combination of no glucose and halted perfusion does result in reduced NVU barrier function and mitochondrial membrane potential.

Similarly, when perfusion was continued, chemical hypoxia alone did not strongly affect NVU function in the tested assays. This may be explained by several factors. The concentration of antimycin used in this study likely does not result in a full inhibition of the electron transport chain [98]. In addition, endothelial cells rely primarily on glycolysis for their energy production rather than mitochondrial respiration [99] and are therefore less likely to be affected by inhibition of the electron transport chain. While endothelial cells rely primarily on glycolysis, energy metabolism in astrocytes and neurons remains subject to debate [100]. While neurons contain many mitochondria and likely rely heavily on mitochondrial respiration [101], it is hypothesized that upon hypoxic conditions, they can switch to glycolysis as a primary source of ATP production: a less efficient, but faster alternative [100, 102, 103]. This may underly our finding showing increased ATP on the brain side of the NVU on a chip model upon exposure to antimycin-A only. Overall, we found that the combination of low glucose, chemical hypoxia, and halted perfusion resulted in impaired NVU barrier function, reduced mitochondrial potential, and lower ATP levels.

Further studies may investigate other components of the complex cascade of events that follows ischemic stroke, such as excitotoxicity, production of reactive oxygen species (ROS), and reperfusion injury [104, 105]. Presence of excess glutamate causing excitotoxicity [106, 107] may be investigated in the NVU on-a-chip model using a fluorescent calcium indicator or by determining glutamate levels in medium samples taken from the brain side of the chip. ROS production [108] can be studied in the NVU on-a-chip model using fluorescent or luminescent assays, or by measuring the ROS contents of apical and basal medium samples. Reperfusion injury [109, 110] may be studied by removal of the stroke conditions and addition of glucose-containing medium without antimycin-A and placing the OrganoPlate back on the rocker platform to reintroduce flow.

Many traditional in vitro NVU models do not incorporate fluid flow. However, numerous publications have reported that incorporation of fluid flow in in vitro NVU models is beneficial, showing reduced BBB permeability, decreased cell division, and increased expression of drug and nutrient transporters [36–38, 42, 43, 46, 111, 112]. Although direct in vivo measurements of shear stress in brain vasculature are lacking and vary dependent on local vessel diameter and curvature, it is estimated that capillaries experience shear stress ≥ 6 dyne/cm^2^. Endothelial cells in our NVU on-a-chip model experience shear stress of ~ 1.2 dyne/cm^2^, falling within the range reported for post-capillary venules (1–6 dyne/cm^2^) [113–115]. Furthermore, the flow in the NVU on-a-chip model reported here is of bidirectional nature, unlike cerebral blood flow in vivo, and flow disturbances are associated with diminished vascular health [115]. By using systems employing pumps and syringes, higher shear stress and unidirectional flow can be achieved in the NVU on-a-chip model presented here. However, this will come at the cost of ease of use and throughput, which is undesirable. While the nature of flow in our system may make the model suboptimal for those research questions requiring full control of all aspects of flow, it has been shown to improve cellular differentiation, polarization, junctional organization, and barrier function compared to static culture [51, 52], indicating that bidirectional flow still holds advantages over static culture in in vitro modeling. More importantly, the lack of a complex setup employing pumps and syringes makes this model amenable to routine experimentation and automation.

## Conclusion

The human NVU on-a-chip model described here presents a significant advancement in complexity over traditional BBB- and NVU-models. Furthermore, it addresses several challenges associated with traditional microfluidic models that require specialized equipment, are often cumbersome to use and low in throughput. Under stroke mimicking conditions, the model shows impaired barrier function and mitochondrial membrane potential of the endothelial vessel, as well as reduced ATP in both blood- and brain compartments. The NVU on-a-chip model can be used for fundamental studies of NVU function in disease and investigation of potential restorative therapies. Due to the platform’s relatively high throughput and compatibility with automation, the NVU on-a-chip model holds potential for drug compound screening.

## Supplementary Information

**Additional file 1.** Schematic representation of the neurovascular unit model in the OrganoPlate. (**a**) Picture of the OrganoPlate 3-lane culture platform, comprising 40 tissue culture chips. (**b**) Picture of the bottom of the OrganoPlate, showing several 3-lane chips. (**c**) Procedure for culturing the neurovascular unit model. ECM gel is loaded in the middle lane. Phaseguides (small rims, dark grey) prevent the ECM gel from overflowing into the adjacent perfusion lanes. Immediately after, neurons and astrocytes are seeded in the bottom lane. After 7 days, brain endothelial cells are added to the top lane to complete the model. The model is cultured under perfusion by placing the OrganoPlate on a rocking platform.

**Additional file 2.** Phase contrast images of the neurovascular unit model in the OrganoPlate. (**a**) Images of a representative chip taken at different days of culture. An ECM gel is loaded in the middle lane followed by astrocyte and neuron seeding in the bottom lane at day 0. Astrocytes and neurons form networks from day 1 to day 7. At day 7, brain endothelial cells are added to the top lane. The endothelial cells attach against the ECM gel and form a microvessel under perfusion. (**b**) Representative chip at day 14 of culture, showing a microvessel of brain endothelial cells in co-culture with networks of astrocytes and neurons. (**c**) High magnification image of brain endothelial cells grown against ECM gel. White arrow indicates the endothelial barrier. (**d**) High magnification image of neuron-astrocyte networks in the bottom lane. Scale bars are 100 µm.

**Additional file 3.** Neurons in the NVU model show spontaneous electrophysiological activity. A fluorescent calcium indicator was loaded into the cells of the NVU on-a-chip model at day 14 of culture. Cells show an increase in fluorescence upon calcium influx into the cell, which is associated with neuronal firing. Calcium fluctuations were captured at 0.5 Hz using a fluorescent microscope. (**a**) Images depict low (dark colors) and high (light colors) intracellular calcium and show a changing pattern in cells over time. (**b**) Fluctuations in calcium signal were plotted over time for four randomly selected active neurons.

## Data Availability

The datasets used and/or analysed during the current study are available from the corresponding author on reasonable request.

## Abbreviations

ATP: Adenosine triphosphate
BBB: Blood–brain barrier
BCRP: Breast cancer resistance protein
CAM: Cellular adhesion molecule
ECM: Extracellular matrix
GLUT-1: Glucose transporter 1
HBMEC: Human brain microvascular endothelial cell
iBEC: iPSC-derived brain endothelial cells
ICAM-1: Intercellular adhesion molecule 1
iPSC: Induced pluripotent stem cell
MRP: Multidrug resistance protein
NVU: Neurovascular unit
Oat: Organic anion transporter
P-gp: P-glycoprotein
P_app_: Apparent permeability
PDMS: Polydimethylsiloxane
PECAM-1: Platelet endothelial cell adhesion molecule
ROS: Reactive oxygen species
s100β: Astrocytic marker S100 calcium-binding protein B
SGLT: Sodium-dependent glucose transporter
TEER: Transendothelial electrical resistance
TMRM: Tetramethylrhodamine methyl ester
TfR: Transferrin receptor
tPA: Tissue plasminogen activator
TUBB3: Beta-III-tubulin
VE-cadherin: Vascular endothelial cadherin
ZO-1: Zona occludens 1
