# Supplementary material for: Modeling ischemic stroke in a triculture neurovascular unit on-a-chip
Source: Fluids Barriers CNS. 2021 Dec 14;18:59. doi: 10.1186/s12987-021-00294-9 (PMC8670153; doi:10.1186/s12987-021-00294-9)
Supplement: Supplementary file 1 — Additional file 1. Schematic representation of the neurovascular unit model in the OrganoPlate. (a) Picture of the OrganoPlate 3-lane culture platform, comprising 40 tissue culture chips. (b) Picture of the bottom of the OrganoPlate, showing several 3-lane chips. (c) Procedure for culturing the neurovascular unit model. ECM gel is loaded in the middle lane. Phaseguides (small rims, dark grey) prevent the ECM gel from overflowing into the adjacent perfusion lanes. Immediately after, neurons and astrocytes are seeded in the bottom lane. After 7 days, brain endothelial cells are added to the top lane to complete the model. The model is cultured under perfusion by placing the OrganoPlate on a rocking platform. [file 12987_2021_294_MOESM1_ESM.pptx]

## Slide 1
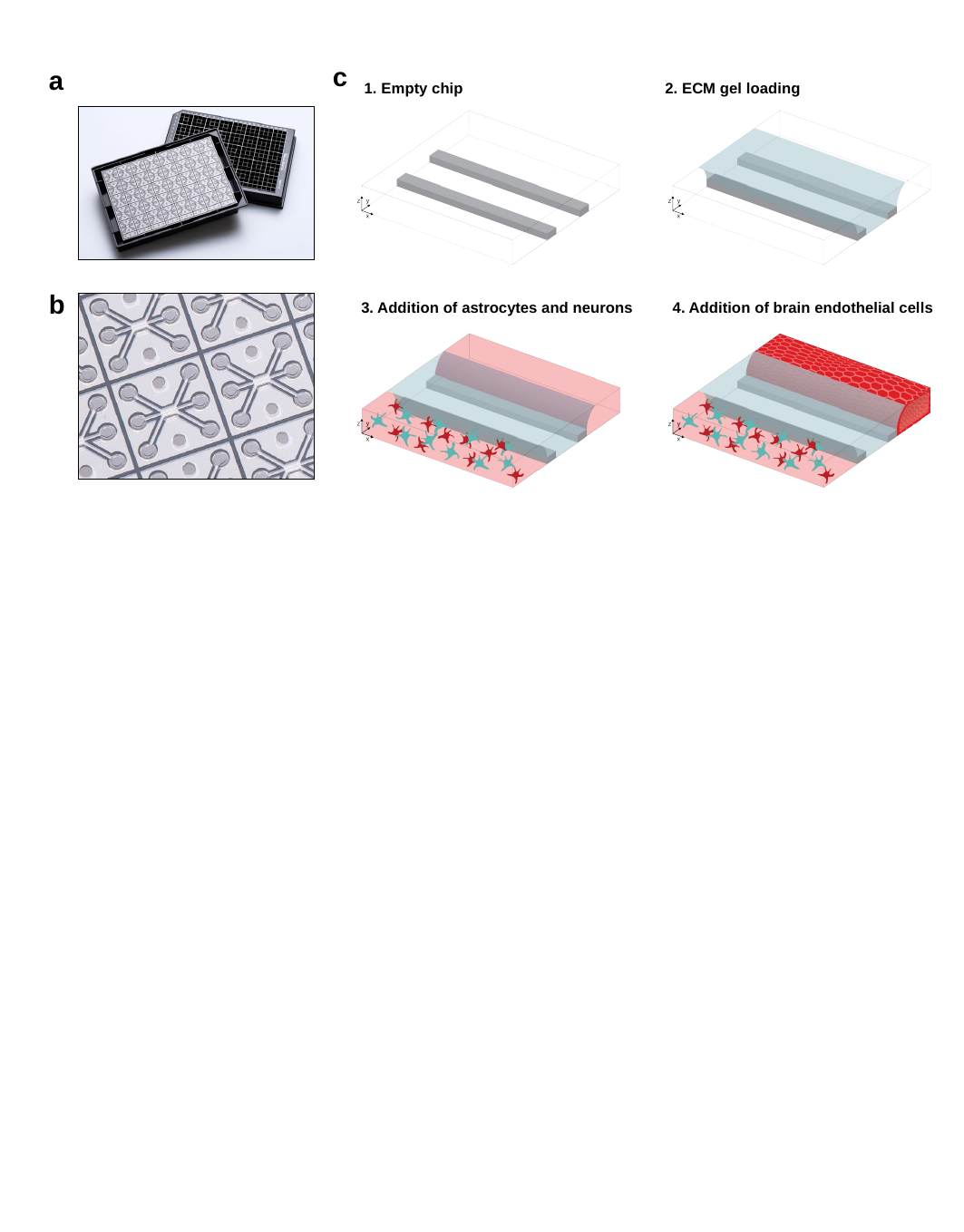

c
a
1. Empty chip
2. ECM gel loading
b
3. Addition of astrocytes and neurons
4. Addition of brain endothelial cells
