# Supplementary figures and images for: Modeling ischemic stroke in a triculture neurovascular unit on-a-chip
Source: Fluids Barriers CNS. 2021 Dec 14;18:59. doi: 10.1186/s12987-021-00294-9 (PMC8670153; doi:10.1186/s12987-021-00294-9)

## Slide 1
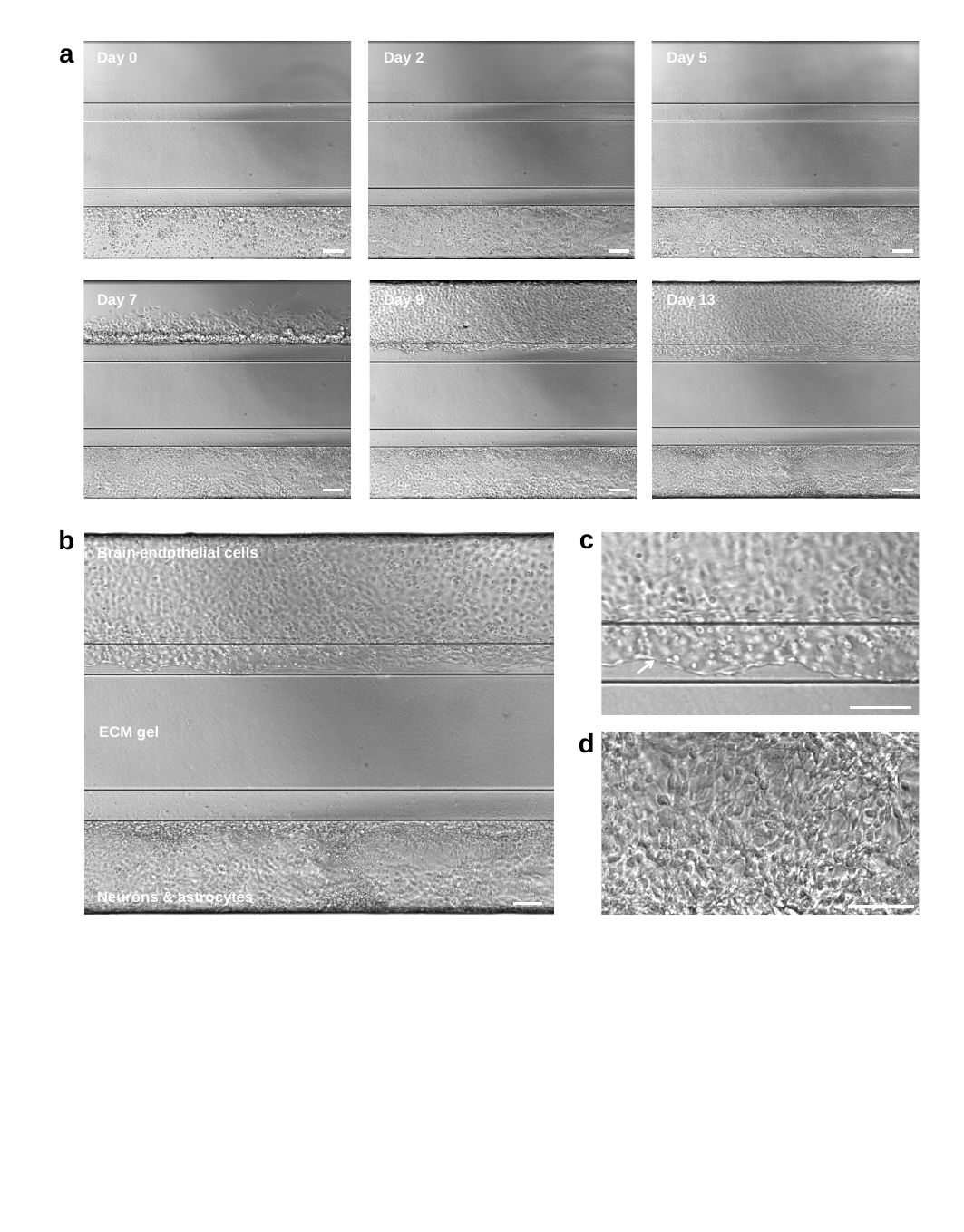

a
Day 0
Day 2
Day 5
Day 7
Day 9
Day 13
c
b
Brain endothelial cells
ECM gel
d
Neurons & astrocytes

Supplement: Supplementary file 2 — Additional file 2. Phase contrast images of the neurovascular unit model in the OrganoPlate. (a) Images of a representative chip taken at different days of culture. An ECM gel is loaded in the middle lane followed by astrocyte and neuron seeding in the bottom lane at day 0. Astrocytes and neurons form networks from day 1 to day 7. At day 7, brain endothelial cells are added to the top lane. The endothelial cells attach against the ECM gel and form a microvessel under perfusion. (b) Representative chip at day 14 of culture, showing a microvessel of brain endothelial cells in co-culture with networks of astrocytes and neurons. (c) High magnification image of brain endothelial cells grown against ECM gel. White arrow indicates the endothelial barrier. (d) High magnification image of neuron-astrocyte networks in the bottom lane. Scale bars are 100 µm. [file 12987_2021_294_MOESM2_ESM.pptx]

## Slide 1
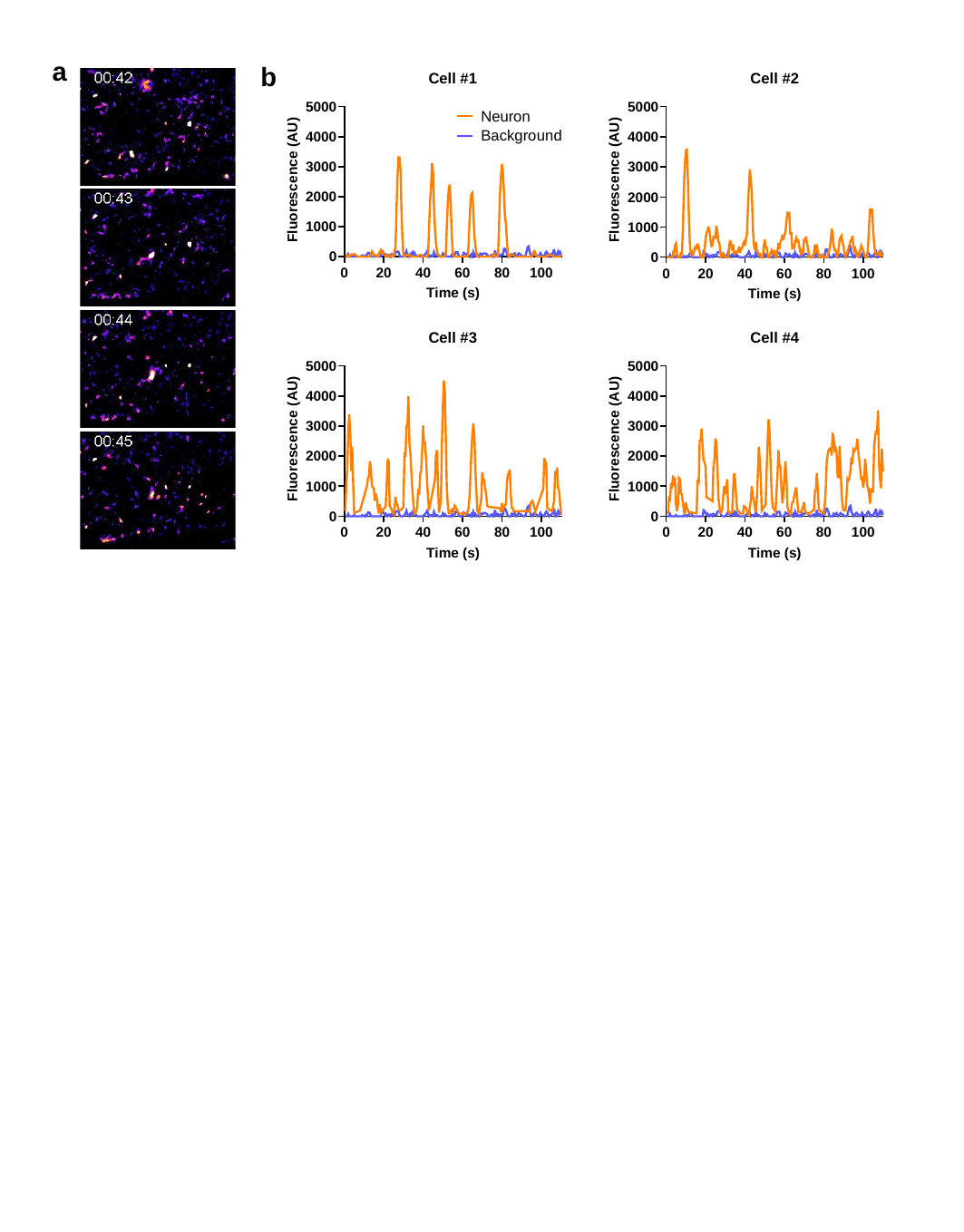

a
b

Supplement: Supplementary file 3 — Additional file 3. Neurons in the NVU model show spontaneous electrophysiological activity. A fluorescent calcium indicator was loaded into the cells of the NVU on-a-chip model at day 14 of culture. Cells show an increase in fluorescence upon calcium influx into the cell, which is associated with neuronal firing. Calcium fluctuations were captured at 0.5 Hz using a fluorescent microscope. (a) Images depict low (dark colors) and high (light colors) intracellular calcium and show a changing pattern in cells over time. (b) Fluctuations in calcium signal were plotted over time for four randomly selected active neurons. [file 12987_2021_294_MOESM3_ESM.pptx]
